# Supplementary material for: Pharmacometrics to Evaluate Dosing of the Patient-Friendly Ivermectin CHILD-IVITAB in Children ≥ 15 kg and <15 kg
Source: Pharmaceutics. 2024 Sep 7;16(9):1186. doi: 10.3390/pharmaceutics16091186 (PMC11435260; doi:10.3390/pharmaceutics16091186)
Supplement: Supplementary file 1 [file pharmaceutics-16-01186-s001.zip › pharmaceutics-3152433-supplementary.pdf]

Supplementary Materials: Pharmacometrics to Evaluate Dosing of the Patient-Friendly Ivermectin CHILD-IVITAB in Children ≥ 15 kg and <15 kg

Klervi Golhen, Michael Buettcher, Jörg Huwyler, John van den Anker, Verena Gotta, Kim Dao, Laura E. Rothuizen, Kevin Kobylinski and Marc Pfister

Figure S1. Goodness-of-fit plots for PK model presented in **Table 2** for ivermectin concentrations by formulation. **A.** Observed ivermectin concentrations (mg/L) versus population predicted ivermectin concentrations (µg/L). **B.** Population-weighted residuals (PWRESs) versus time after dose. **C.** Individual weighted residuals (IWRESs) versus time after dose. **D.** Normalized prediction distribution errors (NPDEs) versus time after dose. Pre-dose samples of next dosing (after wash-out period of 7 days) from cross-over trial were included in the GOFs. Light grey circles correspond to censored data (BLQ, with LLOQ = 0.05 mg/L).

CHILD-IVITAB	STROMECTOL®

A.

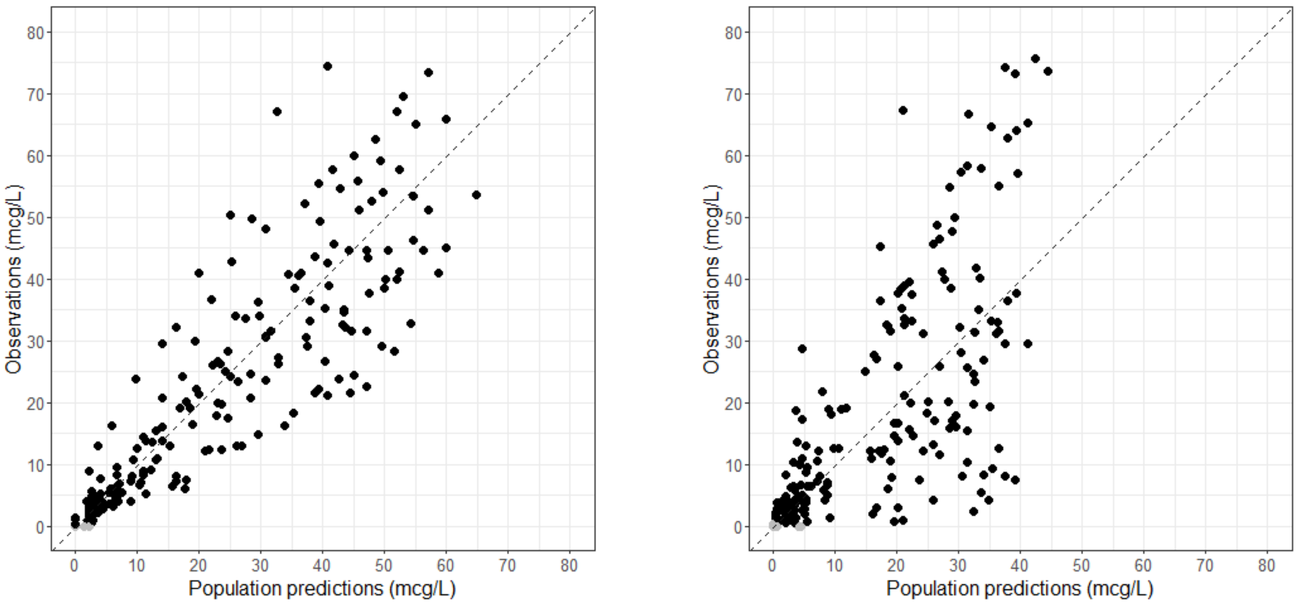

B.

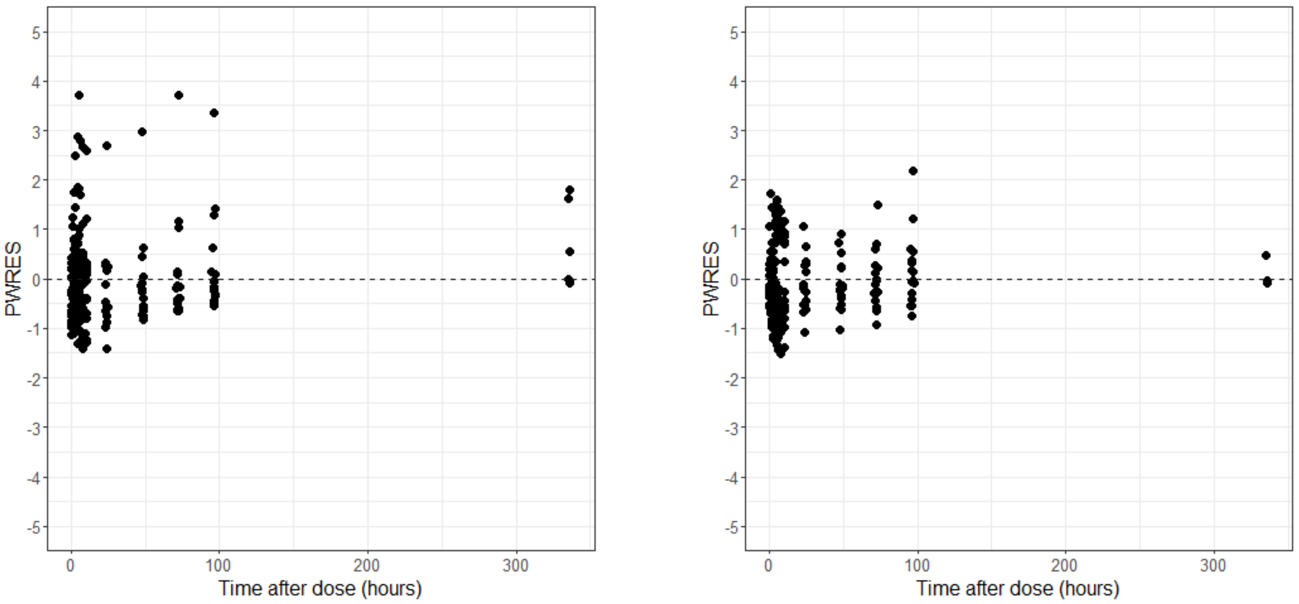

C.

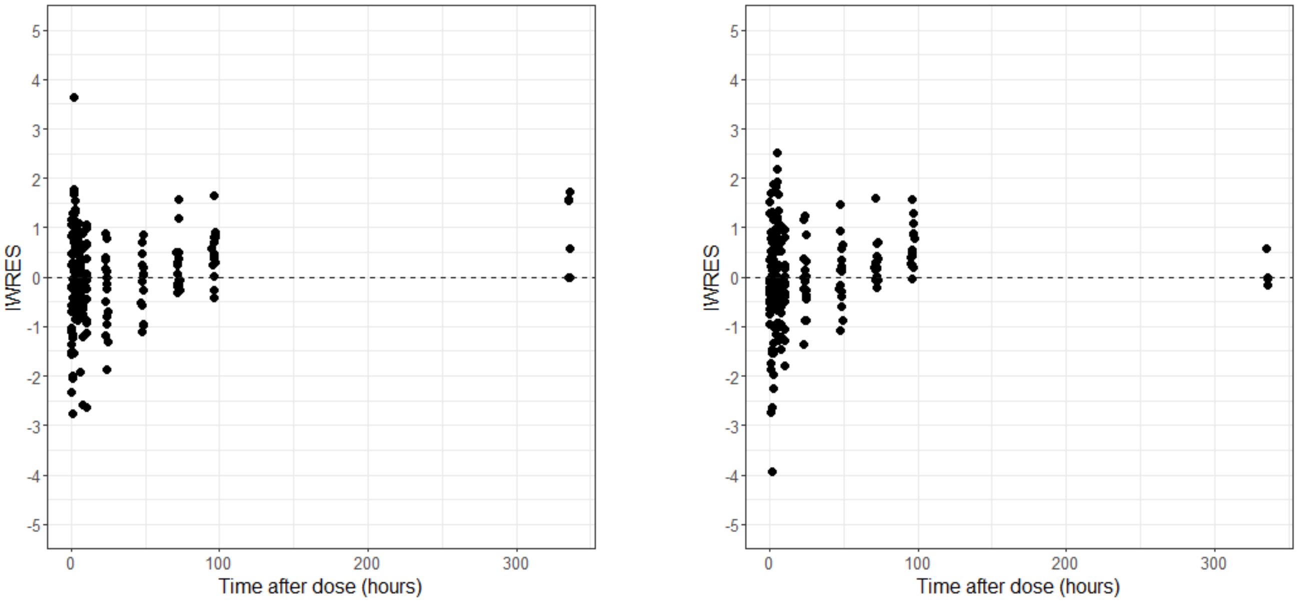
IWRES
Time after dose (hours)
IWRES
Time after dose (hours)

D.

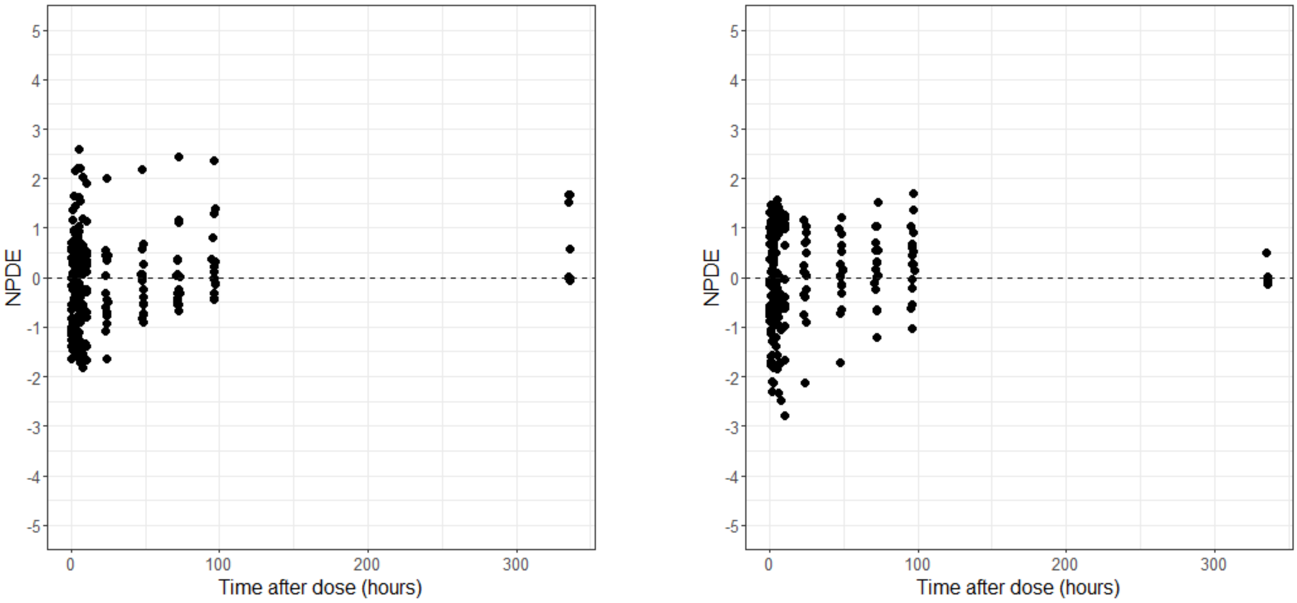
NPDE
Time after dose (hours)
NPDE
Time after dose (hours)

Figure S2. Individual fits (grey lines) of ivermectin concentrations (ng/mL) on y-axis and time after dose (hour) on x-axis. Black dots correspond to observed data. Note: one screening failure occurred (individual #10) because of positive cannabis drug screen.

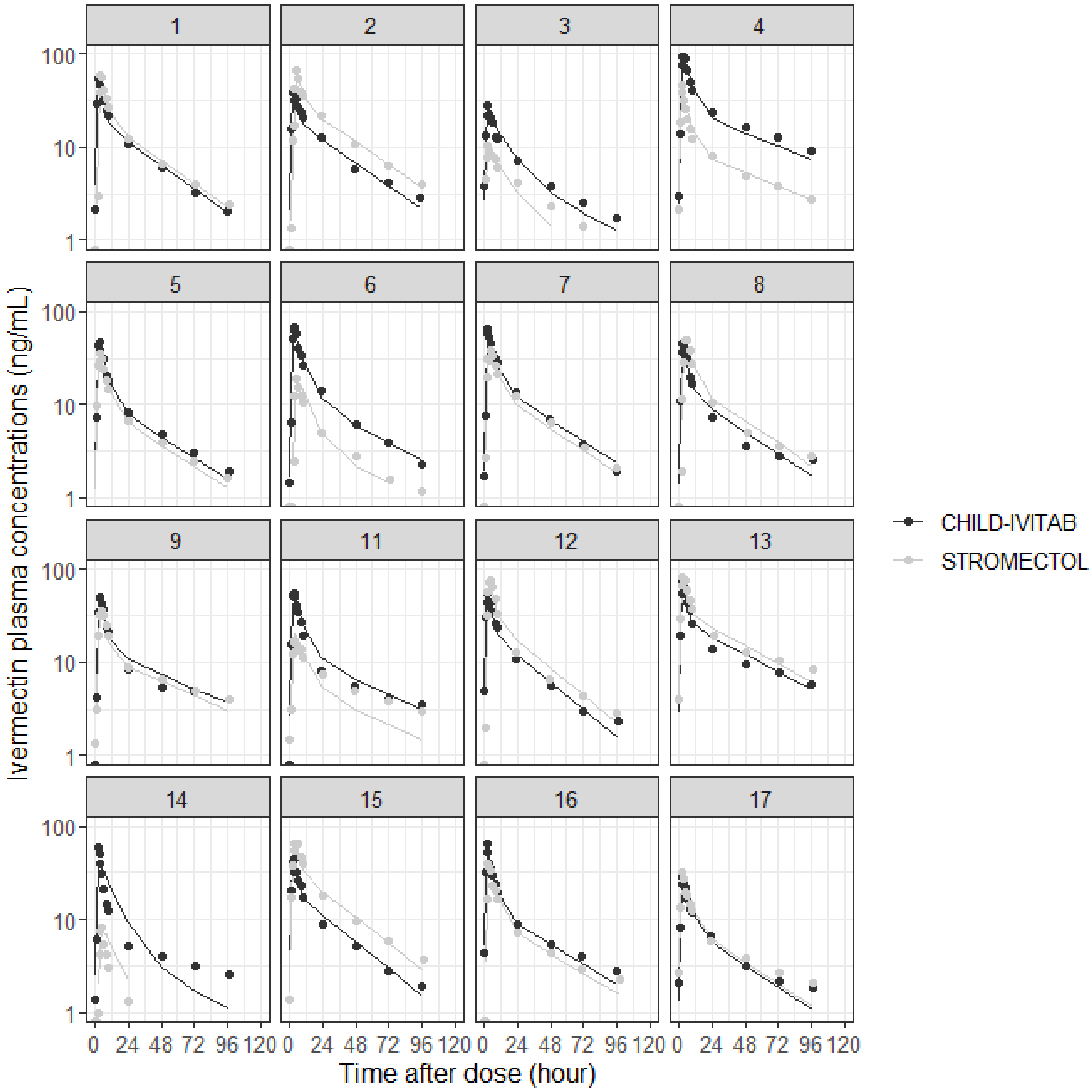

Figure S3. Zoom on absorption phase. Individual fits (grey lines) of ivermectin concentrations (ng/mL) on y-axis and time after dose (hour) on x-axis. Black dots correspond to observed data. Note: one screening failure occurred (individual #10) because of positive cannabis drug screen.

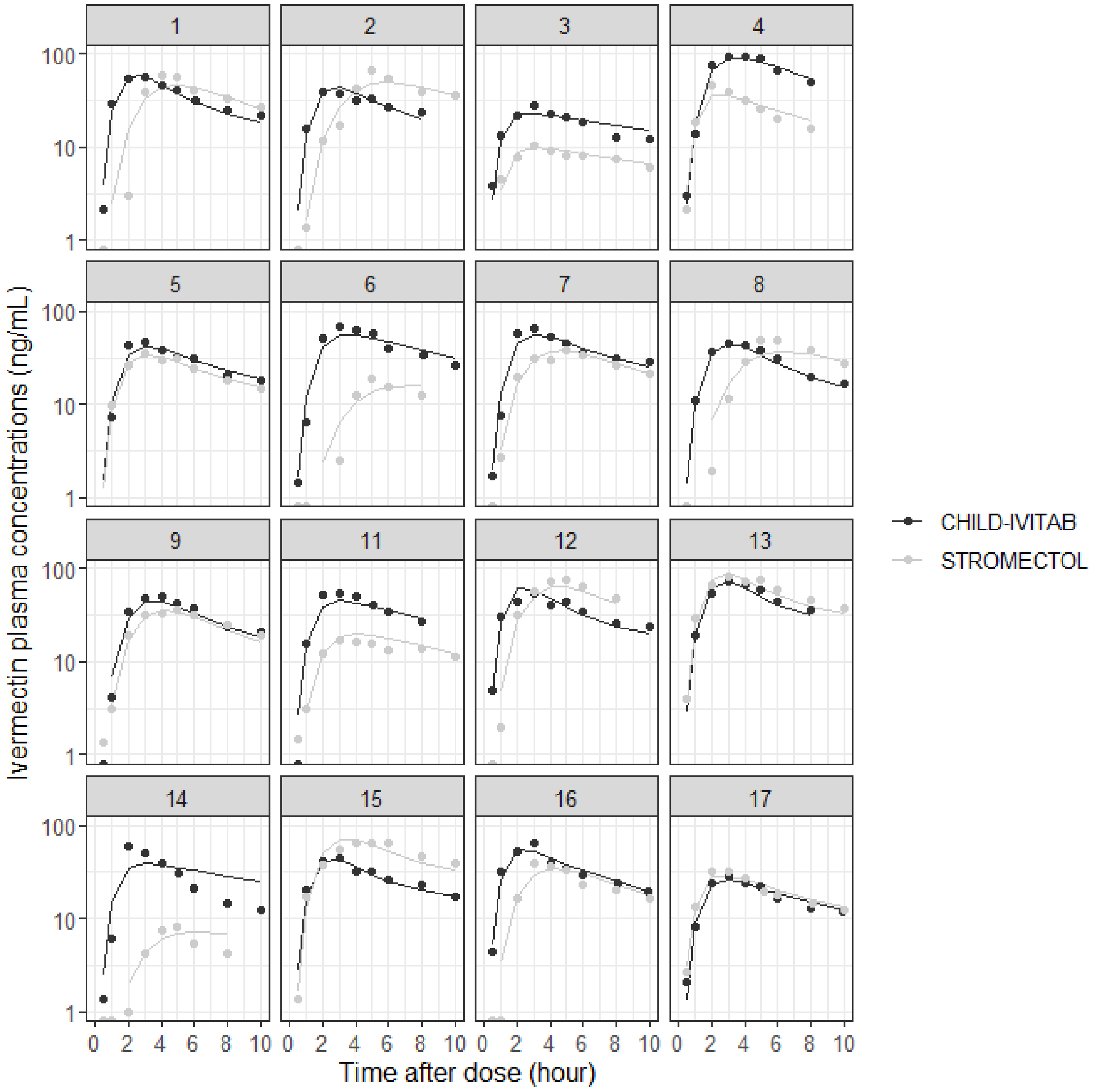

Figure S4. Individual parameter (conditional mode, i.e., empirical Bayesian estimates, EBEs) distribution, represented as histograms for the probability density function. Theoretical individual parameter distribution is represented as continuous black line. Shrinkage value of each individual parameter is displayed on top of the histograms. ka_0, absorption rate constant of CHILD-IVITAB; ka_1, absorption rate constant of STROMECTOL®; CL, clearance; V1, volume of distribution in the central compartment; V2, volume of distribution in the peripheral compartment; relF1, relative bioavailability.

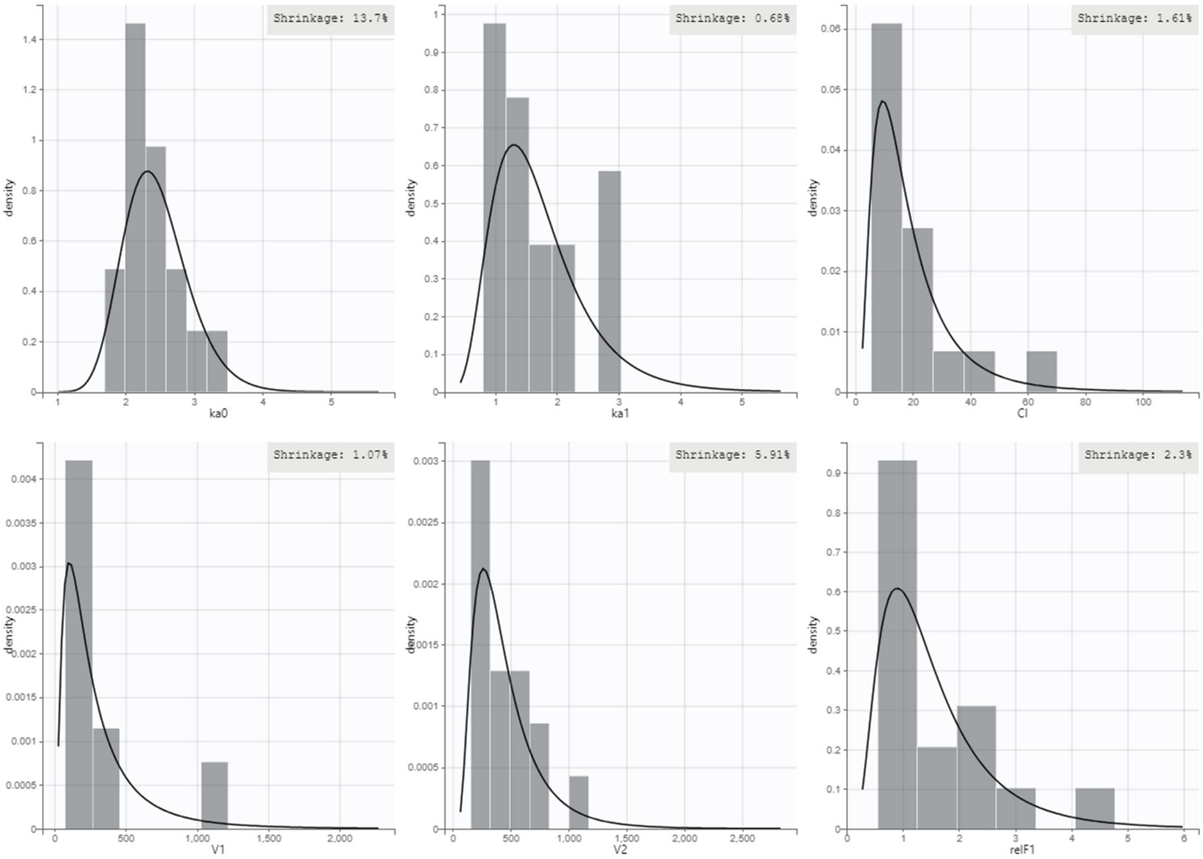

Figure S5. – Simulated ivermectin exposures (N = 1000) 96h ($AUC_{0\text{-}96h}$) and 168h ($AUC_{0\text{-}168h}$) after dosing according to weight-based ivermectin dosing regimen in adults following a single administration of 200 µg/kg of the reference STROMECTOL® formulation and 200, 250 or 300 µg/kg in children <15 kg and ≥ 15 kg following a single CHILD-IVITAB administration.

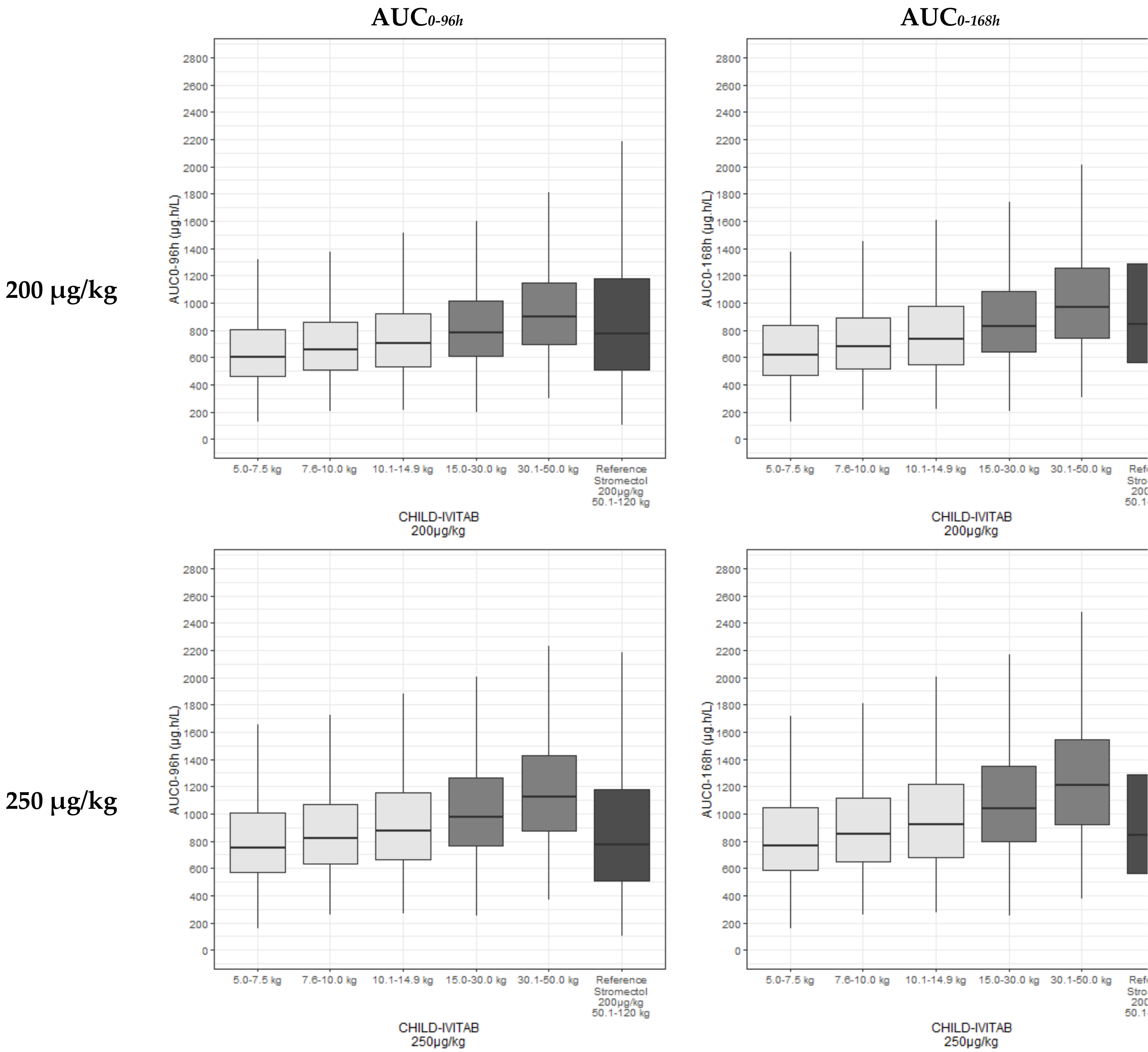

300 µg/kg

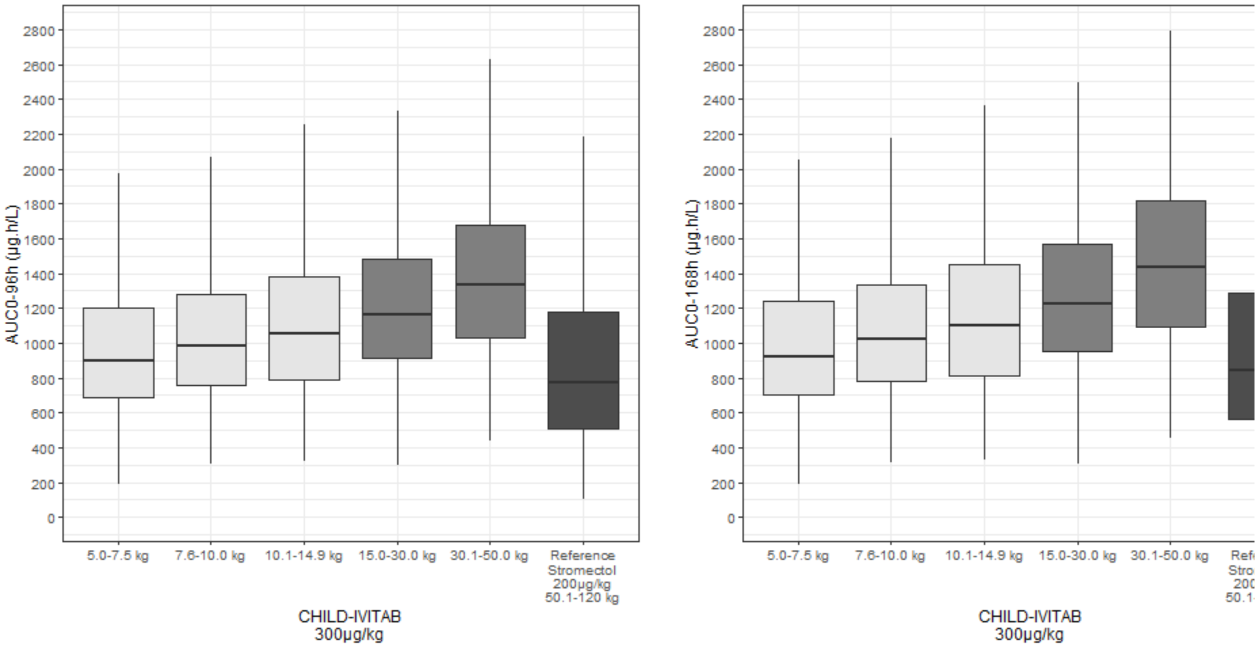

Table S1. Comparison between de novo parameter estimates and parameters from referenced model by Brussee et al. CI, confidence interval; Vc, central volume of distribution; Vp, peripheral volume of distribution.

**Parameter (unit)**		**De novo parameter estimate: median (90% CI)**	**Brussee et al. Bootstrap median (90% CI)**
Absorption rate constant and transit rate constant (h^{-1})	$k_a = k_{tr}$	CHILD-IVITAB: 2.41 (2.20-2.63)	0.904 (0.846-0.967) + 44% in adults [1.30 (1.22-1.39)]
		STROMECTOL®: 1.56 (1.32-1.85)	
Clearance (L/hour)	*CL*	5.8 (4.43-7.58)	5.94 (5.38-6.46)
Volume of distribution (L)	V_c<br>V_p	60.29 (43.02-84.50)<br>103.56 (80.73-132.86)	115 (104-128)<br>90.6 (75.5-130)
Intercompartmental clearance (L/hour)	*Q*	9.73 (8.64-10.97)	5.83 (4.66-7.83)

Table S2. Individual parameter estimates (empirical bayes estimates): CL, clearance; Frel, relative bioavailability; IQR, inter-quartile range.

ID	CL (L/h)	Frel fraction
1	11	0.92
2	7.83	0.61
3	47.09	2.29
4	13.3	2.64
5	19.44	1.23
6	27.54	2.65
7	14	1.28
8	12.18	0.82
9	12.53	1.19
11	22.57	2
12	9.02	0.73
13	5.64	0.8
14	65.83	4.07
15	7.87	0.55
16	16.98	1.31
17	21.7	0.92
Median (IQR)	13.65 (10.51, 21.92)	1.21 (0.82, 2.07)
min, max	5.64, 65.83	0.55, 4.07

Table S3. Objective function value (OFV) according to number of transit compartments per formulation.

Number of transit compartments		OFV
**CHILD-IVITAB**	**STROMECTOL®**	
2	2	2341 (base PK model)
3	2	2325
4	2	2324
5	2	2330
6	2	2338
2	3	2324
2	4	2321
2	5	2325
2	6	2333
3	3	2303
4	4	2294
5	5	2301
6	6	2313

Table S4: Population PK parameter estimates for ivermectin, for base population PK model and sensitivity analysis (excluding subject #14). Proportional and additive errors are reported as variance estimates (σ^2). CL, clearance; Frel, relative bioavailability (Frel set to 1 for the reference formulation STROMECTOL® and estimated Frel for CHILD-IVITAB); IIV, inter-individual variability, reported as coefficient of variation (CV%); Q, intercompartmental clearance; k_a, absorption rate constant; k_{tr}, transfer rate constant; RSE, relative standard error; Vc, volume of distribution in the central compartment; Vp, volume of distribution in the peripheral compartment.

Parameter (unit)		Value (CI: 95%)	
		**Base popPK model**	**Sensitivity analysis (excluding subject #14)**
CHILD-IVITAB absorption rate constant and transit rate constant (h^{-1})	$k_a = k_{tr}$	2.41 (2.17-2.68)	2.42 (2.17-2.69)
STROMECTOL® absorption rate constant and transit rate constant (h^{-1})		1.56 (1.27-1.92)	1.61 (1.30-2.00)
Clearance (L/hour)	*CL*	5.80 (4.21-7.98)	5.09 (3.93-6.59)
Volume of distribution (L)	V_c<br>V_p	60.29 (40.32-90.14)<br>103.56 (76.96-139.35)	54.71 (38.45-77.85)<br>99.59 (75.27-131.78)
Intercompartmental clearance (L/hour)	*Q*	9.73 (8.44-11.22)	8.90 (7.82-10.12)
CHILD-IVITAB relative bioavailability	*Frel*	1.30 (0.97-1.74)	1.18 (0.91-1.52)
IIV $k_{a\ \text{CHILD-IVITAB}}$ (CV%)		0.19 (0.13-0.28)	0.19 (0.13-0.29)
IIV $k_{a\ \text{STROMECTOL}}$ (CV%)		0.43 (0.30-0.60)	0.45 (0.32-0.63)
IIV CL (CV%)		0.67 (0.48-0.95)	0.51 (0.36-0.74)
IIV V_c (CV%)		0.84 (0.60-1.19)	0.71 (0.49-1.01)
IIV V_p (CV%)		0.57 (0.38-0.85)	0.51 (0.34-0.78)
IIV Frel (CV%)		0.61 (0.43-0.86)	0.51 (0.36-0.73)
Additive error (mg/L)		0.69 (0.54-0.84)	0.70 (0.54-0.85)
Proportional error		0.16 (0.14-0.18)	0.15 (0.13-0.16)

Supplementary materials S10: Model code

DESCRIPTION:

The administration is extravascular with a first-order absorption (rate constant ka) with transit compartments (mean transit time, Mtt; transit rate, Ktr).

The PK model has a central compartment (volume V1), a peripheral compartment (volume V2), intercompartmental clearance (Q), and a linear elimination (clearance, Cl).

```
[LONGITUDINAL]
input = {ka0, ka1, Cl, V1, Q, V2, relF1, TREATMENT}
TREATMENT = {use = regressor}
EQUATION:
odeType = stiff
PK:
; Parameter transformations
V = V1
k12 = Q/V1
k21 = Q/V2
if (TREATMENT == 1)
        ka = ka1
  F1 = 1
else
        ka = ka0
  F1 = relF1
end
Ktr = ka
Mtt = 3/Ktr
; PK model definition
Cc = pkmodel(Ktr, Mtt, ka, V, Cl, k12, k21, p=F1)
Ccscaled = Cc * 1000
```

OUTPUT:

output = {Ccscaled}
